# *Botrytis cinerea* and Table Grapes: A Review of the Main Physical, Chemical, and Bio-Based Control Treatments in Post-Harvest

**DOI:** 10.3390/foods9091138

**Published:** 2020-08-19

**Authors:** Nicola De Simone, Bernardo Pace, Francesco Grieco, Michela Chimienti, Viwe Tyibilika, Vincenzo Santoro, Vittorio Capozzi, Giancarlo Colelli, Giuseppe Spano, Pasquale Russo

**Affiliations:** 1 Department of the Sciences of Agriculture, Food and Environment, University of Foggia, Via Napoli 25, 71122 Foggia, Italy; nicola_desimone.552001@unifg.it (N.D.S.); giancarlo.colelli@unifg.it (G.C.); giuseppe.spano@unifg.it (G.S.); pasquale.russo@unifg.it (P.R.); 2 Institute of Sciences of Food Production, National Research Council of Italy (CNR), c/o CS-DAT, Via Michele Protano, 71121 Foggia, Italy; bernardo.pace@ispa.cnr.it; 3 Institute of Sciences of Food Production, National Research Council of Italy (CNR), Via Prov.le Lecce-Monteroni, 73100 Lecce, Italy; francesco.grieco@ispa.cnr.it; 4 InResLab Scarl, Contrada Baione, 70043 Monopoli, Italy; m.chimienti@inreslab.org; 5 AgroSup Dijon, 21079 Dijon CEDEX, France; viwetyibilika@gmail.com; 6 A.B.A. Mediterranea s.c.a.r.l., Via Parini, 1, 74013 Ginosa, Italy; enzo.santoro@abamediterranea.it

**Keywords:** table grapes, *Botrytis cinerea*, grey mould, spoilage microbes, post-harvest, modified atmosphere packaging (MAP), ozone (O_3_), antimicrobial compounds, preservatives, biocontrol

## Abstract

Consumers highly appreciate table grapes for their pleasant sensory attributes and as good sources of nutritional and functional compounds. This explains the rising market and global interest in this product. Along with other fruits and vegetables, table grapes are considerably perishable post-harvest due to the growth of undesired microorganisms. Among the microbial spoilers, *Botrytis cinerea* represents a model organism because of its degrading potential and the huge economic losses caused by its infection. The present review provides an overview of the recent primary physical, chemical, and biological control treatments adopted against the development of *B. cinerea* in table grapes to extend shelf life. These treatments preserve product quality and safety. This article also focuses on the compliance of different approaches with organic and sustainable production processes. Tailored approaches include those that rely on controlled atmosphere and the application of edible coating and packaging, as well as microbial-based activities. These strategies, applied alone or in combination, are among the most promising solutions in order to prolong table grape quality during cold storage. In general, the innovative design of applications dealing with hurdle technologies holds great promise for future improvements.

## 1. Introduction

Viticulture is one of the major forms of fruit crop cultivation worldwide, and its global diffusion contributes considerably to human nutrition. The fruit has a non-climacteric character with a quite low rate of physiological activity. Grapes (*Vitis vinifera* L.) are essential not only for wine production but also for fresh consumption. Table grapes are highly appreciated by consumers, primarily because of their sensory attributes, but also because of their vitamins and bioactive compounds (e.g., flavonoids) [1]. More than 27 million tons of table grapes are produced worldwide annually (an increase of 71% since 2000), and about 4.2 million tons were exported among countries in 2014 [2]. Accordingly, increasing attention has been paid to lengthening the shelf-life of table grapes for export. Prolonged storage time preserves marketability and adds value; however, it is often associated with a decrease in overall product quality. In general, several factors, including bunch dehydration, rachis browning, peel colour changes, lacerations and colonization by various spoilage fungi result in significant economic losses.

**Figure 1:**
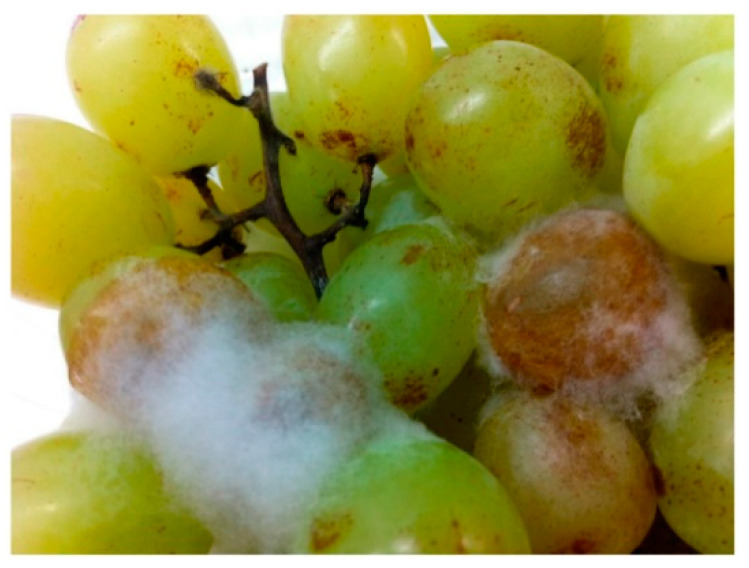
Effect of grey mould on cold-stored cv. “Italia” table grape berries. Image from Ahmed et al. [3].

Among other factors, fungal decay represents the principal factor responsible for post-harvest deterioration in table grapes [3]. *Botrytis cinerea* is the main biological cause of post-harvest problems since it is accountable for grey mould formation [4]. Indeed, this undesired fungus is ranked second in the “world top 10 fungal pathogens in molecular plant pathology” in terms of economic and scientific relevance, preceded only by *Magnaporthe oryzae* [5]. Fungal spores are generally present on the surface of fruits, and, during post-harvest handling the berries can supply a suitable environment for spore germination (mainly the damaged fruits) (Figure 1).

Moreover, the infection can occur during storage, marketing, and even after customer purchase. In the vineyard, high relative air humidity and low environmental temperatures reduce the host’s defences. This environment favours the rapid spread of contamination from a single berry to the whole bunch [6,7]. During post-harvest treatments of fruits and vegetables, processing technologies and biotechnologies provide physical, chemical, and biological hurdles to limit the development of undesired microorganisms [8]. Changes in technical and technological solutions, consumer needs, and regulatory framework lead to a continuous evolution of the handling procedures to limit decay induced by spoilage fungi. All of these advances are generally tailored to reducing and averting spoilage growth, but they are more broadly oriented towards optimization of global quality of production, including safety, health properties, and sensory acceptability [9,10,11,12].

**Figure 2:**
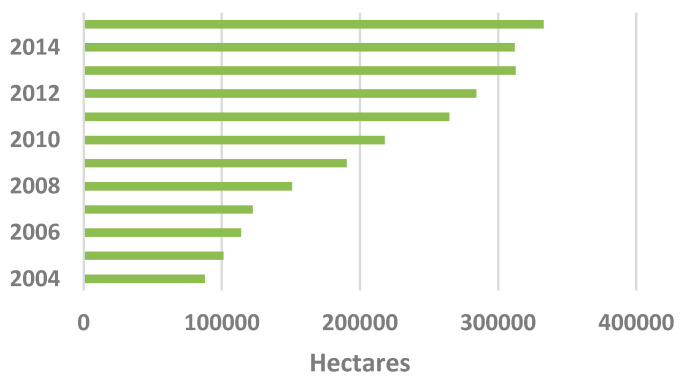
Global area for the cultivation of organic grapes in the period 2004–2015. Source: Research Institute of Organic Agriculture (FiBL) and IFOAM—Organics International—SOEL magazine (2006–2017).

Among the economic and social trends, attention to sustainable viticulture and organic production represents a field of high interest, as evidenced by the rising number of cultivated hectares worldwide (Figure 2).

Nowadays, this kind of table grape cultivation is still increasing in diffusion and economic importance [13]. The production of organic grapes necessitates compliance with specific regulations that limit the chemicals allowed during production and distribution [14]. In general, organic-labelled products are defined as those from plantations that respect and exploit biodiversity, organic turnovers, and soil structure [14]. The European Union has led the cultivation of organic grapes globally, followed by China, the United States of America, and Turkey [15]. Within Europe, the countries with the most extensive acreages dedicated to organic farming are Spain and Italy (1.9 and 1.4 million hectares, respectively; both contributing more than 100,000 hectares to the increase in organic land observed in Europe) [15].

In recent years, different strategies have been proposed to control *B. cinerea* in order to improve the management of post-harvest decay in table grapes and to prevent quality losses [16,17,18]. The present review aims to discuss the more recent investigations conceived to control *B. cinerea* decay in table grapes, including the primary physical, chemical, and biological approaches.

## 2. Physical Methods to Control *B. cinerea* in Table Grapes

Physical technologies mainly include modification of several parameters such as temperature, absolute and relative gas pressure, UV irradiation, and sonication. Table grapes for fresh consumption often need a long period of storage for commercial purposes such as export and ready-to-eat. They are usually stored in chambers with strictly controlled temperature and humidity. To this aim, cold storage (~0 °C) is the primary method to avoid post-harvest infections without affecting the main physicochemical features of the product [19]. However, *B. cinerea* survives at low temperatures, and any variation of temperature can promote water condensation, thus favouring fungal growth and sporulation [20]. In general, physical methods are often considered eco-friendly and residue-free emerging technologies, widely accepted by consumers. Although these methods have been extensively investigated in different fruit and vegetable products, only a few studies report their employment for the reduction of grey mould in table grapes (Table 1).

Surface sanitation is the main strategy implemented to control microbial contamination of fruits and it can be achieved by using different methods. Among these, dipping in hot water (about 50 °C) is an interesting option to prolong the shelf-life of fruits and vegetables [33,34]. Treatments at 50 °C for 10 min, or at 55 °C for 5 min, are sufficient to reduce the fungal growth, maintaining product quality because it does not alter the grape’s organoleptic profile [21,22]. Accordingly, it allows for the marketability of minimally-processed and ready-to-eat table grapes [21,22]. Nonetheless, more studies are requested to improve the processing conditions, i.e., temperature and time of exposure against *B. cinerea* contamination.

Ultraviolet irradiation (UV) (wavelengths between 10 to 400 nanometers (nm)) and sonication by ultrasound are non-thermal treatments considered simple, reliable, and eco-friendly emerging technologies for lengthening the shelf life of fresh fruits during storage. Ultraviolet irradiation C (UV-C, 10–280 nm) treatment induced a general stimulation of the phenylpropanoid pathway, associated with plant defence mechanisms, leading to an increased resistance to the diseases in artificially inoculated berries [24]. UV-C irradiation is effective, with dosages between 0.125 to 0.5 kJ/m^2^ at a fixed distance of 25 cm [35]. In a recent study, harvested ‘Crimson’ red table grapes were exposed to an increased UV-C intensity (6.0 kJ/m^2^), for two illumination periods of 1 min with a specific distance of 60 cm and then maintained at 20 °C for 24 h, followed by cold storage [24]. Regarding ultrasound application, Bal et al. [23] demonstrated the effectiveness of this treatment at 32 kHz, in a distilled water chamber at 20 °C for 10 min. Their study produced encouraging results in preserving grape quality throughout storage for 60 days. A reduction of decay rate was shown and evaluated by scoring the number of contaminated berries, from 2.8 (water-treated control) to 1.5 (ultrasound treated grapes), in an acceptability scale from 1 to 5 points (1 = no decay; 5 = over 20 decayed berries per bunch in a box of 5 kg grapes). It is essential to underline that, in the last two studies, both UV irradiation and sonication are also compared to treatments which combine physical methods with biological compounds, such as chitosan (an antimicrobial linear polysaccharide derived from chitin) and putrescine (biogenic diamine, a class of compound with relevant biological properties), respectively.

Few studies are reported on the use of high hydrostatic pressure and electrolyzed oxidizing water (EOW), especially on table grapes. Romanazzi et al. [25] investigated the efficiency of hyperbaric treatments at 0.15 MPa for 24 h, on artificially inoculated ‘Italia’ table grapes berries, during simulated shelf-life for three days at 20 °C. A significant reduction of the infected berries (from 49.0 to 30.8 %) and of their lesion diameter (from 8.7 to 7.2 mm) was reported for the treated grapes, when compared to control fruits stored at ambient pressure [25]. Electrolyzed oxidizing water is produced through the controlled electrolysis of sodium chloride solutions. Dipping in EOW [250 ppm total residual chlorine (TRC); pH = 6.3–6.5; ORP = 800–900 mV, 1% NaCl] was adequate to prevent the infection of green table grapes artificially contaminated with *B. cinerea* until one week, showing a decay rate of 2% after ten days of storage at 25 °C [26]. Interestingly, a dipping treatment followed by a daily spray of grapes with EOW prevented the infection until 24 days, showing a daily decay rate of 2% after 26 days of storage at 25 °C [26].

The modification of absolute and relative gas pressure, in association with low temperatures during storage, is an important strategy to enhance the shelf life of fruits and vegetables [36]. The main methods include controlled atmosphere (CA) and modified atmosphere packaging (MAP). CA is defined as an atmosphere different than air, applied to commodities in the storage chamber. MAP involves a change in gas environment in packaged commodities, as a result of respiration (passive MAP) or by the different gas permeability of the packaging (active MAP) [37]. The latter method has received considerable attention because of the possibility of maintaining modifications up to consumption [38,39,40]. In both CA and MAP approaches, the use of different gas composition (e.g., changes in ratio Oxygen (O_2_)/Carbon dioxide (CO_2_)) aims to minimize the metabolic activity and oxidative phenomena, thus reducing the physiological decay caused by aerobic microorganisms (e.g., *B. cinerea*) [36,39]. In table grapes, an atmosphere with different gas composition, including high CO_2_/low O_2_ concentrations [41,42,43], and the addition of O_3_ [42], has the effect of reducing decay. Furthermore, this strategy retards senescence, reduces stem and berry respiration, limits rachis browning, and preserves berry firmness [41,42,43]. However, CO_2_ concentrations >10% reportedly promote off-flavor development, rachis and berries’ browning [43]. CA with ozone (O_3_) at 0.3 μL/L was assessed as the minimum concentration to significantly inhibit decay development, in artificially contaminated berries, up to seven weeks in cold storage [28,44]. Recently, in similar storage conditions, ozone-CA with 0.1 μL/L in the day and 0.3 μL/L at night, was found to effectively reduce grey mould, even after 68 days, with a maximum disease incidence of 2.1%, comparable to weekly SO_2_-fumigated grapes [29]. Passive MAP in micro-perforated polypropylene films, was found to have the highest performance in the decay management of ‘Vittoria’ and ‘Red Globe’ table grapes [30]. Cefola and Pace [32] reported best results on ‘Italia’ table grapes, after 14 days of cold storage and three days of shelf-life, by using MAP with an initial concentration of 10% CO_2_, both in terms of sensory quality preservation and decay control. Considering that the use of massive doses of gas in a single pre-storage application can be defined as a sanitation procedure, we refer the discussion to chemical methods following section.

## 3. Chemical Methods to Control *B. cinerea* in Table Grapes

At present, sulphur dioxide (SO_2_) remains the main method that is used to control the microbial spoilage of post-harvest fruit commodities. The employment of SO_2_ provides long term storage due to its antioxidant, antibacterial, antifungal and anti-browning properties [19,45]. However, excessive residue levels of SO_2_ in berry peels can result in quality deterioration, such as bleached berries, production of off-flavour, or hairline disorder [46,47]. Significant health risks to consumers are also reported due to the emergence of allergies, nausea, respiratory distress and skin rashes [48]. For this reason, the United States Environmental Protection Agency (USEPA) categorized SO_2_ as a pesticide, with maximum tolerance in final products of 10 ppm, and, more generally, sulphur dioxide residuals on table grapes are internationally regulated, including in the European Union [49,50]. Its use is also excluded from certified “organic” grapes [16]. Therefore, several chemical alternatives have been proposed to replace SO_2_ in the restraint of *B. cinerea* in table grapes (Table 2).

The use of conventional synthetic fungicides is generating increasing concern among consumers due to the potential negative effects on human health [61], soil microbiota [62], and on microorganisms beneficial for food and beverage fermentations [63]. Even if the use of conventional synthetic fungicides is forbidden for organic grapes [14], application is widespread to prevent spoilage mould formation in conventional agriculture [64]. Despite the fact that some studies have focused on the positive action of different combinations of synthetic fungicides or bioactive compounds [51], the occurrence of resistant strains of *B. cinerea* has been reported [65]. The most recently introduced class of synthetic fungicides belongs to the Succinate Dehydrogenase Inhibitors (SDHIs) [66]. In 2012, a novel SDHI, named fluopyram, was registered against *B. cinerea* and it was able to control grey mould infections in table grapes, with efficacy of inhibition in the range 80.1–94.4% [52]. However, high risks of rapid occurrence of resistance without appropriate management has already been underlined in other crops [67]. For this reason, alternative control methods are needed. Among these, resistance induced by elicitors, molecules able to activate defence gene expression and enhance their antimicrobial-related pathways [68], is an attractive alternative because it is associated with minor environmental risk. Acibenzolar-S-methyl is a commercial elicitor able to activate the phenylpropanoid pathway, which leads to the accumulation of lignin, phenolic compounds and flavonoids [68]. In table grapes, it can be used as spray aspersion or dipping solution, both with a significant reduction in terms of decay incidence [53].

Other chemicals are widely used as dipping solutions to sanitize fruit surfaces. The treatment of grapes by immersion or spraying with solutions of different generally recognized as safe (GRAS) salts at 1% reduced the percentage of spoiled fruit. This was the case with iron sulphate (FeSO_4_) (92%), ammonium bicarbonate (NH_4_HCO_3_) (91%), sodium silicate (Na_2_SiO_3_) (89%), sodium bicarbonate (NaHCO_3_) (76%) and sodium carbonate (Na_2_CO_3_) (74%) (application in pre-harvest, decay measured post-harvest) [55]. However, treatment with FeSO_4_ could cause small black spots on the grape surface [55]. Disinfection by dipping in 32% ethanol, followed by six weeks of cold storage, reduced natural decay incidence on ‘Scarlotta Seedless’ from about 60% to 4.1% [54]. Nevertheless, the use of large quantities of ethanol is expensive and may be dangerous, due to its flammability. A more practical method is the use of ethanol vapour-generating bags, that confer longer protection, effectively reducing decay incidence in artificially inoculated grapes stored for one month, in a comparable way to SO_2_ generating-pads in polyethylene bags [56]. In this case, significantly lower weight loss and moderate stem browning were also observed [56]. Furthermore, it is relevant to underline that active coatings associated with selected films represent a promising strategy to increase table grape shelf life [69].

Recently, Gorrasi et al. [70] demonstrated the efficacy of active packaging based on a food grade acrylic resin filled with Layered Double Hydroxide (LDH) nanofiller hosting antimicrobial 2-acetoxybenzoic anion (salicylate), on microbial control during table grape (cv Egnathia) storage.

In addition to ethanol vapours, other gas types have been used as fumigation treatment for the sanitization of bunches. With this scope, chlorine dioxide (ClO_2_) is a gaseous disinfectant admitted in the sanitization of uncut and unpeeled fruits and vegetables. In a recent study, Chen et al. [57] reported a reduction of decay incidence and of rachis browning in table grapes treated with ClO_2_ during storage. The Food and Drug Administration (FDA) has approved ClO_2_, given that these treatments might leave chlorite residues on food products at non-hazardous concentrations [71]. Nitrous oxide (N_2_O) is another gas tested to control post-harvest decay in fruit crops. In vitro tests did not show inhibition against grey mould; however, in vivo experiments in table grapes fumigated for 6 h showed a significant reduction in decay development during six days of cold storage [58]. Therefore, it was hypothesized that N_2_O was indirectly able to inhibit grey mould by increasing the host′s disease resistance [58].

The use of pre-treatments with high concentrations of CO_2_ have been widely studied; these showed great potential in decay control and prevention of water loss and oxidative damage [59]. In Cardinal table grapes, these effects seem to be related to the specific induction of defence proteins, including dehydrins and proteins associated with pathogenesis, as well as endogenous protective osmolytes [59]. In the last few years, different concentrations of CO_2_ were evaluated. Pre-treatments with 20% of CO_2_ for three days [59], 40% CO_2_ for 48 h followed by CA storage [27], and 50–70% for 24 h followed by MAP [31], were all effective against post-harvest decay of the cultivars assayed. Although all the treatments guaranteed basic quality standards for commercial table grapes, a concentration-dependent effect has been observed. However, as previously mentioned, the use of pre-storage application of a high concentration of CO_2_ causes cultivar-dependent collateral effects such as rachis, berries browning and off-flavours [43].

Ozone fumigation is one of the most prominent sanitation strategies for fruits and vegetables [72,73]. Different approaches have been developed for ozone-based treatments on table grapes [74,75]. Among these, continuous exposure in controlled atmosphere during cold storage has been reported [28,29]. Decay reduction was confirmed only with pre-treatment at 20 μL/L for 30 min, followed by MAP storage [31]. Interestingly, intermittent ozone treatment (2 μL/L, 12 h for day) induced higher resveratrol accumulation (in three different table grape cultivars) [60]. Moreover, this could be responsible for decreases in the level of pesticide residues (phenomena reported for grapes stored in ozone atmosphere) [75,76]. Nevertheless, ozone is corrosive and represents a worker hazard [77], and, among the quality parameters, significant weight loss during storage was usually highlighted [28,44,60].

## 4. Biological Methods to Control *B. cinerea* in Table Grapes

Consumers widely accept the development of bio-based applications to exert microbial control in agro-food chains because of the growing demand for eco-friendly approaches and products free of synthetic chemicals [78,79,80]. For these purposes, several protective cultures [81,82,83,84] and compounds of biological origin [80,85] have been assessed for their possible use as Biological Control Agents (BCAs) against *B. cinerea* in table grapes.

### 4.1. Microbial Resources

Several yeast species are found in association with the surface of the grapes, in particular, the genera *Saccharomyces*, *Candida*, *Dekkera*, *Pichia*, *Hanseniaspora*, *Metschnikowia*, *Kluyveromyces*, *Saccharomycodes*, *Schizosaccharomyces*, *Torulaspora*, and *Zygosaccharomyces* [86,87]. Highly variable in terms of relative proportion, often as a function of the sanitary condition of the grapes, these species have different significances in oenology, i.e., pro-technological, spoilage, biocontrol, production of toxic catabolites [88,89,90,91,92]. On the other hand, it is possible to find prokaryotic organisms present on the grape surface that exert their biotechnological action in the last phases of the winemaking process [93]. This broad microbial diversity justifies massive isolation of yeasts and bacteria to preserve and characterize strains of biotechnological interest [94,95,96]. This isolation can be of microorganisms from plants, grape bunches, musts or wines and selection is made of those capable of inhibiting undesired microbe development on grapevines [97,98] up to the final steps of wine production [99]. This reservoir of microbial-based biocontrol solutions has also been exploited in fruits [100,101,102,103], in several cases offering the option to inhibit *B. cinerea* in table grapes (Table 3).

Among yeast species, strains belonging to *Saccharomyces* are the most commonly studied because of their pivotal function in alcoholic fermentation and their role as a biological model organism [117,118,119]. Recently, Nally et al. [108] used a fruit decay test on wounded table grape berries to screen the activity of 65 yeasts, previously tested against *B. cinerea* by using in vitro approaches. They found that 15 *S. cerevisiae* strains and one strain of *Sch. pombe*, isolated from grape must, were able to reduce grey mould decay [108]. Among these, the disease incidence of grapes treated with *Sch. pombe* BSchp67 reached 29.9%, while 9 strains of *S. cerevisiae* were able to fully inhibit decay development when added at a concentration of 10^7^ cells/mL [108].

Regarding the non-*Saccharomyces* yeasts, *H. uvarum* is a species of enological interest, usually present on the grape surface [120,121]. In various studies, it has demonstrated an antagonistic property, mainly based on competition for living space [122]. The addition of this yeast has been implicated in the reduced incidence of grey mould disease in artificially inoculated table grapes [111]. Moreover, this antagonistic activity was enhanced by the addition in the formulation of salicylic acid or salts, such as sodium bicarbonate or ammonium molybdate [109,123]. *Starmerella bacillaris* (synonym *Candida zemplinina*) is another species of interest, commonly isolated from grapevines/musts [124,125] and from wines fermented by using botrytized grapes [126,127]. Three *Starm. bacillaris* strains, recently isolated from these wines, denoted a significative antifungal activity, probably addressable to the release of volatile organic compounds (VOCs) [110]. The production of VOCs is widely diffused among yeasts. Mewa-Ngongang et al. [112] observed a fungicidal effect of *C. pyralidae* Y1117 and *P. kluyveri* Y1125, mediated by VOC release in a closed environment, able to inhibit fungal growth for five weeks of storage [112].

In vivo studies demonstrated that grey mould can be efficiently controlled by various microbial antagonists isolated from a large variety of vegetal matrices. *Wickerhamomyces anomalus* BS91, *M. pulcherrima* MPR3, and *Aureobasidium pullulans* PI1 were isolated from spontaneous olive fermentation and pomegranate, minimally processed. In detail, *M. pulcherrima* strain showed the best antifungal activity (disease incidence (DI) = 6.7%, disease severity (DS) = 2.7%), followed by *W. anomalus* BS91 and *A. pullulans* PI1, and all of these yeasts were capable of VOC production [106]. In particular, the antagonistic activity of *W. anomalus* seemed to be connected to a killer phenotype [106]. Enzyme secretion in the environment, such as b-1,3-glucanase, pectinase, and protease, was also reported for *W. anomalus* and *A. pullulans* [106], whereas, the activity of *M. pulcherrima* was probably associated with iron depletion [128]. In the patenting literature, two patents based on *M. fructicola* strain’s biocontrol applications for viticultural applications have been reported [129].

Epiphytic *Issatchenkia terricola* yeasts isolated from ‘Thompson Seedless’ grapes’ surface have shown the ability to reduce decay caused by *B. cinerea* up to 80% compared to the untreated control [104]. In another study, yeast and bacteria strains were isolated from fruits and leaves of the same cultivar without any signs of infection, and tested for potential applications in biocontrol [107]. Yeasts were identified as *Candida membranifasciens* Kh69 and *Meyerozyma guilliermondii* Ka21 and Kh59, while bacteria were *Bacillus* spp. Kh26 and *Ralstonia* spp. N1. All tested microbes were able to increase *B. cinerea* inhibition from 23.8% to 54.7%. Among these, the highest level was found for *Ralstonia* spp. N1(54.7%), while *Bacillus* spp. Kh26 and *M. guilliermondii* Ka21 and Kh59 showed inhibition below 50% [107].

Still on the prokaryotic side, a bacterial strain, *Paenibacillus pasadenensis* R16, isolated from grapevine cultivar ‘Barbera’, has shown a reduction in disease incidence of grey mould by 27.5% [116]. It was also supposed that the main metabolite responsible for antifungal activity was farnesol which was never before reported to have biocontrol potential [116]. A large number of bacterial strains belonging to *Bacillus* spp. are reported to have antimicrobial activity against several plant phytopathogens [130,131,132]. In fact, a lot of commercial bio-fungicides, such as *B. subtilis* QST713 (Serenade^®^, Bayer CropScience) and *B. amyloliquefaciens* FZB24 (Taegro^®^, Novozymes), are now available and effective against grey mould on grapes. Recently, Chen et al. [115] demonstrated the ability of four *Bacillus* strains, isolated from various ecological niches, to control decay development in table grapes and other fruit crops. The most vigorous antifungal activity was recorded in *B. subtilis* Z-14 [115]. VOC production, enzyme, siderophores, and lipopeptide antibiotics were proposed as possible modes of action.

### 4.2. Antimicrobial Compounds of Biological Origin

Recently, there have been intense investigations conducted in the field of natural antimicrobials and their effectiveness. Many biological compounds have been tested for the bio-control of table grape spoilages. These compounds include classes of chemicals/matrices such as vegetal extracts, essential oils, and defence inducers (Table 4).

Among the vegetal compounds, volatiles generated from cellulose soaked with garlic hydro-alcoholic extract and its derived sulfur compounds have shown anti-grey mould activity in packaged table grapes both at 4 and 25 °C, during the 14 days of experimental trials [133]. However, organoleptic and sensorial adverse effects of this treatment have still not been investigated [133]. Cinnamic acid, extracted from cinnamon bark, is widely used as a food additive. Dipping the berries in a solution of 10 mM cinnamic acid can significantly decrease the incidence of decay development up to half of that in control after four days of storage at 25 °C [134]. Hinokitiol is a natural monoterpenoid mainly extracted from the wood of *Cupressaceae*. In a recent study [135], no decay was visible after 60 h at 22 °C in artificially wounded/inoculated table grape berries treated with a 3 g/L hinokitiol solution [135].

Essential oils (EOs) from many plants, such as thymus and lemongrass, have revealed great potential in post-harvest disease control [144]. In addition, the effect of mint EOs was recently investigated by using direct contact (e.g., dipping) and volatile methods (filter paper) [136]. In this study, EO released by the paper was more effective than the direct contact and was capable of inhibiting *B. cinerea* in artificially inoculated trials during nine days of shelf-life [136]. However, the effect on product flavour and consumer acceptance was not investigated.

Another research field involves the use of vegetal hormones, plant activators, and inner signalling molecules. These molecules act through a complex signalling network under the control of salicylic acid, ethylene, jasmonic acid, and phenylpropanoid pathways, which leads to the increase of specific secondary metabolites (e.g., flavonoids, soluble sugars, and phytoalexins). Methyl jasmonate is a volatile compound that mediates stress responses in plants and has shown to promote fungal resistance in various fruit crops. Recently, it was found to be effective in lessening the development of *B. cinerea* in artificially infected table grapes [137]. In this study, the fruits were packed in the presence of a filter paper soaked with a solution of methyl jasmonate at 10 µmol/L and stored at 25 °C [137]. The disease incidence in the treated fruits after 24, 36, and 48 h was 41.7%, 60.6%, and 86.5% of that in the control trial, respectively [137].

Fulvic acids (FA) are the soluble fraction of natural organic matter and are used in agriculture as a plant growth promoter and to control several plant diseases. Xu et al. [138] assayed different concentrations of FA as dipping solutions for wounded table grape fruits, subsequently sprinkled with a conidia suspension of *B. cinerea*. After six days of incubation at 22 °C, the treatment with a solution at 20 mg/mL FA was found to be effective by reducing decay development [138]. The authors suggested that secondary metabolites produced by the berry mediate antifungal activity. However, the formation of necrotic spots was reported [138].

Among secondary metabolites, phytoalexins are synthesized by the plants as broad-spectrum inhibitors. Stilbenoids, including pterostilbene and piceatannol, are phytoalexins commonly found in vine leaves and wine [139]. “Mare’s milk” table grapes treated with 50 mg/L pterostilbene did not show any sign of infection while piceatannol at the same concentration reduced grey mould disease by 75% after nine days storage at 22 °C [139]. These molecules seemed to be the most effective in a group of seven phenolic compounds, including resveratrol and coumarin [139].

Edible coatings made with natural polymers like chitosan or alginate can act as a cover material able to wrap the berry. Thus, these formulations can extend the shelf-life of fruit crops and maintain quality reducing water losses [145,146]. Chitosan is a linear polysaccharide composed of D-glucosamine and N-acetyl-D-glucosamine linked by a β-(1→4) bond obtained by treating the exoskeleton of arthropods with alkaline solutions. Recently, it was found that chitosan-silica nanocomposite polymers can reduce the incidence of decay in grape berries by 59% [140]. Moreover, this coating can be used to incorporate bioactive compounds. An additive effect of chitosan combined with *Salvia fruticosa* Mill. extract [141] and *Mentha piperita* or *M. villosa* essential oil [142] was reported. Alginate is another biocompatible and biodegradable polymer extracted from brown algae and used as a food additive with the code E401. It was demonstrated that the incorporation of vanillin, a phenolic compound, in a coating formulation prolongs the shelf life of table grapes until 35 days of storage, by reducing total yeasts and mould counts [143]. However, the retention of soluble solids, titratable acidity, firmness, and color was also enhanced.

## 5. Conclusions and Future Directions

Post-harvest fungal decay of fruits and vegetables is responsible for huge levels of economic loss and account consistently for large quantities of agro-food waste [147,148,149,150]. To improve economic, social, and environmental sustainability in the sector of table grapes, this review paper provides an overview of the wide plethora of physical, chemical, and bio-based solutions to improve the control of fungal pathogens and spoilage fungi. Each treatment has peculiar benefits and limitations that affect the concrete applications and shape different future perspectives [151]. For example, considering limitations, ozone does not always penetrate natural openings efficiently; condensation inside the package (MAP) increases the chance of microbial decay of produce; the antagonistic target of a biocontrol agent can have a strain-dependent spectrum. In some cases, the limitation is due to lack of harmonization of regulations and consumer acceptance (e.g., irradiation), and investment needs compared to the volume of production (e.g., CA storage) rather than of specific technological or biological issues [151].

As in other fields of food technology, an integrated management program (combining two or more different solutions) could be useful to minimize post-harvest losses caused by undesired fungal development [147,152,153,154,155]. Synergistic approaches have also been developed to reduce *B. cinerea* incidence in table grapes, adopting hurdles technology [23,24,27,31,51]. In other cases, one treatment aimed to reduce microbial contamination, while another was applied to stabilize fruit quality and the microbial population during cold storage and/or shelf-life [27,31,156]. Moreover, it is important to underline that a consistent range of solutions has been developed and tested on other fruits and vegetable [157,158,159,160,161,162,163] and, in several cases, could be tested/transferred for application on table grapes. Among the other green solutions, poorly explored in grapes, is the exploitation of lactic acid bacteria as biocontrol agents [164,165]: prokaryotic organisms that received interest also in the light of additional positive side effects, e.g., probiotic activity and antagonistic activity against food-borne pathogens [166,167,168,169,170].

## Figures and Tables

**Table 1:** Main physical methods investigated in the last ten years against grey mould decay in table grapes.

Physical Methods	Treatment Intensity	Cultivar	Effects	Ref.
Hot Water Treatments	Dipping for 5 min at 55 °C	Müşküle and Red Globe	Low decay rate after three weeks of cold storage; sensory evaluation results showed no alteration of flavor and taste	[21]
	Dipping for 10 min at 50 °C	Crimson Seedless	Inhibition the microbial growth during storage without significant changes in texture, titratable acidity, and soluble solids content	[22]
Ultrasound	32 kHz at 20 °C for 10 min	Michele Palieri	Combined with putrescine, the treatment maintained high levels of anthocyanins, total phenolic content, antioxidant capacity, sensory acceptability and reduced decay incidence during storage	[23]
UV-C Irradiation	Two times at 6.0 kJ/m^2^ for 1 min at 60 cm	Crimson	Combined with chitosan coating, the treatment increased the resveratrol content, maintained sensorial quality, and reduced fungal decay	[24]
High Pressure	0.15 MPa for 24 h at 20 °C	Italia	Reduction of lesion diameter and decay rate after three days of shelf-life	[25]
Electrolyzed oxidizing water	(250 ppm TRC; pH = 6.3–6.5; ORP = 800–900 mV, 1% NaCl) dipping and daily spray	Thompson seedless	Prevention of infection until seven days; 1% of incidence and 2% of severity were reported after 10 days of shelf-life at 25 °C	[26]
CA	12% O_2_ + 12% CO_2_	Flame Seedless and Crimson Seedless	Combined with CO_2_, the treatment limited decay incidence in both naturally and artificially infected grapes	[27]
	0.3 μL/L O_3_	Sultanina	Reduction of fungal decay during 40 days of cold storage; no significant alteration of quality characteristics	[28]
	0.1 - 0.3 μL/L O_3_	Crimson Seedless	Reduction of natural incidence of decay by approximately 65% after five–eight weeks of storage.	[29]
MAP	Passive modifications packaging-induced	Vittoria and Red Globe	Reduction of weight losses, rachis and berry decay	[30]
	2% O_2_ + 5% CO_2_	Scarlotta	Combined with O_3_, the treatment was efficient in decay control but caused sensorial quality losses (intense stem browning, off-flavors perception) Combined with CO_2_, the treatment controlled the concentration of acetaldehyde, preserved rachis chlorophyll content and skin color; also, cumulative decay incidence was reduced	[31]
	Initial concentration of 10% CO_2_	Italia	Decay control during 14 days of cold storage, and three days of shelf life, low acetaldehyde, and ethanol accumulation	[32]

**Table 2:** Main chemical methods investigated in the last ten years against grey mould decay in table grapes.

	Molecules	Treatment	Concentration	Cultivar	Effects	Ref.
Liquid	Pyrimethanil	Wound inoculation	50 mg/L	Crimson Seedless	Combined with resveratrol (1 g/L), the treatment reduced disease incidence and lesion diameter	[51]
	Fluopyram	Spraying	250 µg/mL	Italia	Efficacy against fungicide-resistant fungal strains	[52]
	Acibenzolar-S-methyl	Dipping	1% w/v	Italia and Benitaka	Reduction of grey mould development after one month of cold storage and one week of shelf life, without alteration of the physicochemical quality	[53]
	Ethanol	Dipping	32 %	Scarlotta Seedless	Reduction of berries decay until ten weeks of storage	[54]
	FeSO_4_, NH_4_HCO_3_, Na_2_SiO_3_, NaHCO_3_ and Na_2_CO_3_	Dipping or spraying	1% w/v	Benitaka	Decay incidence reduced, no impact on berries quality parameters with minor exceptions which were at an acceptable level	[55]
Gas	Ethanol	Vapour-generating bags	-	Red Globe	Comparable to SO_2_ treatments in decay control, the treatment enhanced berry colour, but caused stem browning	[56]
	Chlorine dioxide (ClO_2_)	Injection in bag	2.5 mg/5 kg	Kyoho	Reduction of berry decay and rachis browning	[57]
	Nitrous oxide (N_2_O)	Fumigation	50 μL/L	Munage	Reduction of lesion diameter and decay incidence	[58]
	Carbon dioxide (CO_2_)	Fumigation	20 %	Cardinal	The treatment avoided post-harvest losses in terms of water loss, oxidative damage and disease prevention	[59]
		Fumigation	40%	Flame Seedless and Crimson Seedless	Combined with CA, the treatment limited decay incidence in both naturally and artificially infected grapes	[27]
		Fumigation	50–70%	Scarlotta	Combined with MAP (2% O_2_ + 5% CO_2_), the treatment was efficient in decay control but caused sensorial quality losses (intense stem browning, off-flavours perception)	[31]
	Ozone (O_3_)	Fumigation	20 μL/L	Scarlotta	Combined with MAP (2% O_2_ + 5% CO_2_), the treatment controlled the concentration of acetaldehyde, preserved rachis chlorophyll content and skin colour; the cumulative decay incidence was also reduced	[31]
		Periodic fumigation	2 μL/L	Superior Seedless, Cardinal CL80, and Regina Victoria	The treatment increased resveratrol content but led to low scores in sensory evaluation; high weight loss was also reported	[60]

**Table 3:** Main microbial strains investigated in the last ten years against grey mould decay in table grapes. Where possible, Inhibition Percentage (IP), Disease Incidence (DI), and Disease Reduction (DR) were reported to quantify the activity of each strain.

	Microbial Strain	Source of Isolation	Activity	Cultivar Tested	Ref.
Yeasts	*Issatchenkia terricola* 156a5	Thompson seedless	IP = ~80%	Flame seedless	[104]
	*Wickerhamomyces anomalus* BS91	Fermented olive and pomegranate	DI = ~50%	Not specified	[105,106]
	*Metschnikowia pulcherrima* MPR3		DI = 6.7%		
	*Aureobasidium pullulans* PI1		DI = ~55%		
	*Meyerozyma guilliermondii* Ka21, Kh59	Thompson seedless	IP = 47.6%	Thompson seedless	[107]
	*Candida membranifaciens* Kh69		IP = ~42%		
	*Saccharomyces cerevisiae* spp. (9 strains)	Grape must	DI = 0%	Red globe	[108]
	*Schizosaccharomyces pombe* BSchp67		DI = 29.92%		
	*Hanseniaspora uvarum* SEHMA61	Wild grape	-	Not specified	[109]
	*Pichia kluyveri* SEHMA6B		-		
	*Starmerella bacillaris* PAS151	Ripe grape must	DR = ~40%	Not specified	[110]
	*Hanseniaspora uvarum*	Strawberry	DI = 51,8%	Kyoho	[111]
	*Candida pyralidae* Y1117	Grape must	DI = 0%	Regal seedless	[112,113]
	*Pichia kluyveri* Y1125	*Sclerocarya birrea* juice	DI = 0%		
Bacteria	*Bacillus* sp. Kh26	Thompson seedless	IP = 49.9%	Thompson seedless	[107]
	*Ralstonia* sp. N1		IP = 54.7%		
	*Bacillus amyloliquefaciens* NCPSJ7	Ginger field	DI = 36%	Red globe	[114]
	*Bacillus amyloliquefaciens* RS-25	Jujube fruit	DR = 86.6%	Red globe	[115]
	*Bacillus licheniformis* MG-4	Strawberry	DR = 84.7%		
	*Bacillus subtilis* Pnf-4	Wheat plant	DR = 69.95%		
	*Bacillus subtilis* Z-14	Wheat soil	DR = 42.43%		
	*Paenibacillus pasadenensis* R16	Barbera	DR = 27.5%	Black magic	[116]

**Table 4:** Main biological compounds investigated in the last ten years against grey mould decay on table grapes.

	Biological Compounds	Concentration	Treatment	Cultivar	Effects	Ref.
Vegetal extract	Hydro-alcoholic garlic extract and derived sulfur compounds	2 mL and 20 μL	Volatiles release	Flame Seedless	The treatment efficiently controlled the decay in packed grapes at 4 and 25 °C for 14 days	[133]
	Cinnamic acid	10 mM	Dipping	Manai	The treatment halved the decay incidence after four days at 25 °C	[134]
	Hinokitiol	3 g/L	Wound inoculation	Manai	No visible decay was reported after 60 h at 22 °C	[135]
Essential Oil	Mint EO	500 μL/L	Volatiles release	Not specified	Reduction of decay in packed grapes	[136]
Other compounds	Methyl jasmonate	10 µmol/L	Volatiles release	Kyoho	Reduction of the decay incidence	[137]
	Fulvic acid	20 mg/mL	Dipping	Mare’s milk	Induction of resistance mainly through the activation of phenylpropanoid pathway	[138]
	Pterostilbene and Piceatannol	50 mg/L	Wound inoculation	Mare’s milk	Reduction of disease incidence and severity	[139]
	Putrescine	1–2 mM	Dipping	Michele Palieri	Combined with ultrasound, the treatment maintained high levels of anthocyanins, total phenolic content, antioxidant capacity, sensory acceptability and reduced decay incidence during storage	[23]
Edible coating	Chitosan	-	Coating	Crimson	Combined with UV-C irradiation, the treatment increased the resveratrol content, maintained sensorial quality, and reduced fungal decay	[24]
	Chitosan/Silica polymer	0.5–1%	Spraying	Italia	The treatment reduced natural infection; no adverse effect in terms of quality (titratable acidity [TA], total soluble solids [TSS], berry color, mass loss, stem browning and shattered berries) was observed	[140]
	Chitosan + *Salvia fruticosa* Extract	500 mg/L (SE)	Dipping	Thompson Seedless	Control efficacy comparable to thiabendazole, decreased the weight loss during cold storage, preserved TSS and TA	[141]
	Chitosan + Mint Essential Oil	1.25–5 μL/mL (MEO)	Dipping	Isabella	The treatment delayed the decay development and reduced incidence; color and firmness were enhanced, did not negatively affect TSS and TA	[142]
	Alginate + Vanillin	0.5–1.5% (V)	Spraying	Lavalleé and Razaki	Reduction of natural yeasts and mould growth, prevention of weight and firmness losses. TSS, TA, and color showed minor changes compared to control grapes.	[143]
